# Genomic Analysis of Global *Staphylococcus argenteus* Strains Reveals Distinct Lineages With Differing Virulence and Antibiotic Resistance Gene Content

**DOI:** 10.3389/fmicb.2021.795173

**Published:** 2021-12-02

**Authors:** Cosmika Goswami, Stephen Fox, Matthew Holden, Alistair Leanord, Thomas J. Evans

**Affiliations:** ^1^Institute of Infection, Immunity and Inflammation, University of Glasgow, Glasgow, United Kingdom; ^2^School of Medicine, University of St Andrews, St Andrews, United Kingdom; ^3^Scottish Microbiology Reference Laboratories, Glasgow, United Kingdom

**Keywords:** *Staphylococcus argenteus* pathogenicity islands, *Staphylococcus argenteus*, antibiotic resistance, virulence genes, CRISPR/Cas

## Abstract

Infections due to *Staphylococcus argenteus* have been increasingly reported worldwide and the microbe cannot be distinguished from *Staphylococcus aureus* by standard methods. Its complement of virulence determinants and antibiotic resistance genes remain unclear, and how far these are distinct from those produced by *S. aureus* remains undetermined. In order to address these uncertainties, we have collected 132 publicly available sequences from fourteen different countries, including the United Kingdom, between 2005 and 2018 to study the global genetic structure of the population. We have compared the genomes for antibiotic resistance genes, virulence determinants and mobile genetic elements such as phages, pathogenicity islands and presence of plasmid groups between different clades. 20% (*n* = 26) isolates were methicillin resistant harboring a *mec*A gene and 88% were penicillin resistant, harboring the *blaZ* gene. ST2250 was identified as the most frequent strain, but ST1223, which was the second largest group, contained a marginally larger number of virulence genes compared to the other STs. Novel *S. argenteus* pathogenicity islands were identified in our isolates harboring *tsst-1, seb, sec3, ear, selk, selq* toxin genes, as well as chromosomal clusters of enterotoxin and superantigen-like genes. Strain-specific type I modification systems were widespread which would limit interstrain transfer of genetic material. In addition, ST2250 possessed a CRISPR/Cas system, lacking in most other STs. *S. argenteus* possesses important genetic differences from *S. aureus*, as well as between different STs, with the potential to produce distinct clinical manifestations.

## Introduction

*Staphylococcus argenteus* is a newly described species of bacteria associated with community-associated infection, which was first identified as a divergent clade within the *Staphylococcus aureus* clonal complex (CC)75 (Holt et al., 2011). It was formally classified as a separate species in 2015 based on its genetic signature (Tong et al., 2015). This species shows many phenotypic characteristics with *S. aureus*, and is indistinguishable from *S. aureus* by routine clinical diagnostic methods, such as tests for coagulase or clumping factor. MLST (Multi Locus Sequence Typing) and *crtM* gene absence may provide specific identification (Becker et al., 2019). *S. argenteus* was first reported in a community of northern Australia in 2002 as causing a skin and soft tissue infection (Ng et al., 2009) and gradually it was reported in other tropical countries (Chantratita et al., 2016; Chen et al., 2018; Kitagawa et al., 2020) as well as in Europe (Dupieux et al., 2015; Argudín et al., 2016; Hallbäck et al., 2018), including the United Kingdom. *S. argenteus* was initially suggested to be less virulent than *S. aureus* (Holt et al., 2011) but later studies suggested that infections with *S. argenteus* carry a significantly higher risk of producing respiratory infection, higher morbidity and mortality than methicillin-susceptible *S. aureus* (MSSA) (Chen et al., 2018; Johansson et al., 2019). In addition to skin and respiratory infections, *S. argenteus* is also reported to cause necrotizing fasciitis (Holt et al., 2011), bone and joint infection (Rigaill et al., 2018), bacteremia (Chantratita et al., 2016), and food poisoning (Suzuki et al., 2017). Comparison of the *S. argenteus* dominant ST ST2250 with various *S. aureus* strains isolated in Thailand show distinctive antibiotic profile variation between them. Isolates from Thailand are predominantly ST2250 which is methicillin susceptible but resistant to penicillin (Moradigaravand et al., 2017), whereas sequenced ST2250 isolates from Denmark and United States are often methicillin resistant strains, harboring the Staphylococcal cassette chromosome *mec* type IV (SCCmecIV) (Okuma et al., 2002; Hansen et al., 2017). However, given the non-random selection of strains for sequencing, these patterns may not reflect the overall prevalence of antibiotic resistance within *S. argenteus* in these regions. Another emerging ST of *S. argenteus*, ST1223, which had been reported related to food poisoning (Suzuki et al., 2017; Wakabayashi et al., 2018) was also found to be resistant to methicillin.

Other *S. argenteus* strains have been identified in marginally lower numbers in humans (Thaipadungpanit et al., 2015; Zhang et al., 2016; Aung et al., 2017), a few in livestock (Argudín et al., 2016) and also from wildlife such as the African gorilla and bat (Akobi et al., 2012; Schuster et al., 2017). Virulence genes were mostly carried in mobile genetic elements such as plasmids, pathogenicity islands and bacteriophages (Goerke et al., 2009). Almost all *S. aureus* genomes contain staphylococcal pathogenicity islands (SaPI)-like elements and genomic islands νSaα and νSaβ (Baba et al., 2002). Different types of SaPIs and associated virulence genes have been identified (Novick et al., 2010). In *S. argenteus*, a SaPI-like element SargPID7903 was identified in a Danish isolate harboring staphylococcus exotoxin B gene (*seb*) and a penicillin-binding protein gene *ear* (Hansen et al., 2017). Comprehensive analysis of mobile genetic elements in *S. argenteus* and associated virulence determinants has not been performed. In addition, genomic studies of inter lineage variation within *S. argenteus* have been limited to small numbers of isolates.

*Staphylococcus argenteus* can cause significant human disease, and clearly shares many features in common with the much better understood related species *S. aureus.* Previous studies have explored genetic similarities and differences between these two species with either small numbers (Long et al., 2020) or from one sequence type and geographical location (Moradigaravand et al., 2017). Whole genome sequencing gives a fine-scale insight into bacterial genomes that enables macroevolutionary relationship of lineages to be established and the variety of virulence determinants, mobile genetic elements and antibiotic resistance genes to be determined. The aim of this study was to use global whole genome sequence data to obtain a greater insight into the genetic differences and similarities between *S. argenteus* and *S. aureus*.

## Materials and Methods

### Data Collection and Sequencing

A total of 132 *S. argenteus* genomes were collected from fourteen different countries deposited between the years 2005 and 2018. Of these, 129 genome sequences were collected from National Centre for Biotechnology Information Collection (NCBI) genome database and three United Kingdom isolates were from Whole Genome Shotgun (WGS) sequencing project database PRJEB12513. Out of the 129 genomes from the NCBI database, Illumina short reads were not available but the assembled genome contigs were used for our analysis. Clinical information available for these samples were the sample collection date, source of collection or host (e.g., Human, Pig, Gorilla, etc.) and country of collection; for some studies, source of infection was also available. From the United Kingdom, three genomes were collected from the Reference Laboratory in Glasgow, Scotland from years 2015–2016 and were sequenced at the Wellcome Sanger Institute, Cambridge using the Illumina HiSeq 2000 platform. The sequencing generated 100-bp paired end reads with an average sequencing depth of 66-fold. The sample accession numbers, assembly accession, assembly size and GC% of all the samples used in this study are shown in Supplementary Table 1. Where comparison is made to specific *S. aureus* sequences, their accession numbers are given in the text or figure legend. Publications from the Bioproject studies citing these sequences are as follows: PRJEB9575 (Thailand), (Moradigaravand et al., 2017); PRJEB20633 (Denmark) (Hansen et al., 2017); PRJNA317277 (China) (Zhang et al., 2018); PRJNA305687 (Sweden) (Hallbäck et al., 2018).

### Genome Assembly and Accessory Genes

To identify the STs from the assembled contigs, we have used MLST v2.16.2^1^ with the *S. aureus* database from BIGSdb (Jolley and Maiden, 2010; Maiden et al., 2013). *De novo* assembly of those samples, with available sequences reads, were performed using Shovill v1.0.0^2^ with minimum FASTQ read depth 150, minimum contig coverage 2 and assembler SPAdes for the paired-end reads with k-mer sizes 21, 33, and 55. After filtering small contigs (less than 1000 bp), the assemblies were rearranged according to a reference genome *S. argenteus* 58113 (Acc. No. AP018562.1) using Mauve v2015-02-13 genome aligner (Darling et al., 2010) for a complete alignment of the conserved regions among the input genome sequences with initial seed-size log2(average sequence length) and minimum recursive gap length as 200. These genomes were further annotated for genes with Prokka v1.2 (Seemann, 2014) with genus Staphylococcus and *e* value 1e-9. The pan-genome of these sequences were identified with Roary (Page et al., 2015) allowing paralog gene splits for a minimum 95% identity in protein database BLAST and a threshold of 99% of isolates for a gene to be in core. Non-metric multidimensional scaling (NMDS) and gene accumulation curve was performed using the ‘‘vegan’’ package in R^3^. For NMDS “metaMDS” function was used to calculate the “Jaccard” distance matrix between genomes with k = 2 and trymax = 100. The Gene accumulation curve was calculated using “specaccum” function from “vegan” package with classic “random” method for 100 permutations. For pathway identification between different STs, DAVID geneontology (Huang et al., 2008a,b) helped to classify the functional variation between the STs.

### SNPs and Phylogenetic Analysis

For phylogenetic analysis, ParSNP v1.2 (Treangen et al., 2014) was used to filter the core genome SNPs from the assembled sequences using 1000 as the maximum distance between two colinear MUMs. The *S. argenteus* 58113 strain (ST2250, Acc No. AP018562.1) was used as the reference for variant calling. Phages were masked from the reference genome after identifying them with PHASTER web-server (Zhou et al., 2011; Arndt et al., 2016). From this reference-based SNP-alignment, recombination regions were identified using Gubbins (Croucher et al., 2015) for a threshold of minimum of three SNPs to identify a recombination and for maximum of 5 iterations for tree convergence under the “recombination” method of convergence. The Maximum Likelihood SNP based phylogeny for 132 samples was inferred using RAxML (Stamatakis, 2015) with the generalised time reversible (GTR) model and a gamma distribution to model site-specific rate variation for 1000 bootstraps. The isolates were also checked for a temporal signal using TempEst v1.5.3 (Rambaut et al., 2016) that gives the linear regression of the root to tip distance of the population. The best fit of the root to tip divergence for each of the isolates gave a correlation coefficient of 0.36 and a very low R^2^ of 0.13. Determination of a temporal signal was not possible due to the absence of raw sequence reads of some of the isolates.

### Antibiotic Resistance and Virulence Genes

For identification of antibiotic resistance genes, the database ARG-annot2 (Gupta et al., 2014) was used for nucleotide BLAST (Altschul et al., 1990)search against the contigs. This database includes genes mediating resistance against the following antibiotic classes: aminoglycosides, beta-lactamases, fosfomycin, fluoroquinolones, glycopeptides, macrolide-lincosamide-streptogramin, phenicols, rifampin, sulfonamides, tetracyclines, and trimethoprim. If an alignment of at least 90% nucleotide sequence identity was detected that covered at least 90% of the gene length, the ABR gene was considered present in the genome. Similarly, Plasmidfinder (Carattoli and Hasman, 2019) database was used for plasmid replication gene identification using nucleotide BLAST search with 90% minimum identity and 85% minimum coverage. Virulence gene identification was done by using database VFDB (Chen, 2004) with the same cut off as plasmids. This database curates all sequences that enable a microorganism to establish itself on or within a host of a particular species and enhance its potential to cause disease, including bacterial toxins, cell surface proteins that mediate bacterial attachment, secretion system components, cell surface carbohydrates and proteins that protect a bacterium, and hydrolytic enzymes that may contribute to the pathogenicity of the bacterium. Statistical analysis on virulence gene variation between the STs was performed using R and significant values were calculated by two-sample Mann-Whitney test, adjusted with Bonferroni corrections in R.

### SaPI Island Detection and Phages

To identify the SaPI-like elements, SaPI integrases were extracted from whole SaPI islands of 31 fully identified SaPI references (Novick et al., 2010; Sato’o et al., 2013; Suzuki et al., 2015)using Artemis Genome visualization (Carver et al., 2011). These integrases were used to perform nucleotide BLAST against the contigs for >90% identity and checked for the alignment lengths. The whole SaPI islands were also searched using nucleotide BLAST to investigate for the island similarity for >60% coverage threshold. EasyFig (Sullivan et al., 2011) was used to plot the similarity between different SargPI islands. Phage integrases from each integrase group (Goerke et al., 2009) were also extracted using Artemis. We defined phage groupings based on the phylogenetic relationships of their integrases, as described (Goerke et al., 2009). Phage1 included Sa2, Sa4, and Sa6 integrases; Phage2 contained Sa3, Sa8, and Sa9 integrases; Phage3 consisted of Sa1 and Sa5 integrase group and Phage4 had Sa12 and Sa7 integrase groups.

### Restriction Modification System

For the analysis of the hsdS amino-acid phylogeny, sequences were aligned using MAFFT (Katoh et al., 2002) and a maximum likelihood phylogeny created using RAxML (Stamatakis, 2006) using automatic selection of best amino-acid substitution model by setting the -m flag to PROTGAMMAAUTO and using a rapid bootstrap calculation (-f flag set to a) and 1000 bootstrap calculations. FigTree^4^ was used to visualize the resultant best tree. To show homologies between different hsdS protein sequences, representative aligned sequences were visualized using the function msaplot in the ggtree package (Yu et al., 2017). Aligned sequences were viewed in Jalview (Waterhouse et al., 2009) to determine Blosum 62 overall homology scores across aligned sequences (Henikoff and Henikoff, 1992; Eddy, 2004).

### CRISPR-Cas Loci Identification

The identification of signature genes for CRISPR-Cas types and subtypes were searched by the CRISPRCasFinder web-server (Couvin et al., 2018) on the assembled genomes. For each CRISPR array size of flanking regions was set for 100 bp; repeats were identified with repeat length threshold (min. 23 and max 55) and for spacers minimum 60% sequence similarity was taken. The Cas genes were detected using Prodigal and MacSyFinder program by HMM search on the Cas protein library (Abby et al., 2014). A total of 1021 CRISPR were identified along with some small CRISPR-like by-products within our genomes. Discarding these false positives, a collection of 175 CRISPRs were obtained (with evidence level > 1) and a pool of 44 unique spacers were extracted from these CRISPRs. These spacers were then grouped for 90% similarity using cd-hit^5^ to give 24 spacer groups named SCP1--SCP24 having an average length of 37 bp (ranging from 33 bp till 58 bp). Nucleotide based Maximum likelihood tree (with 1000 bootstraps using RAxML as described above) was built to identify the phylogenetic relationship between the spacer sequence representatives and highly similar sequence searches were carried out in the National Center for Biotechnology Information (NCBI) nucleotide database^6^, using BLASTn for bacterial genomes, plasmids and bacteriophages.

*fos* gene analysis. Selected *fos* genes were aligned using SEAVIEW (Gouy et al., 2009) with the CLUSTAL omega (Sievers and Higgins, 2018). A maximum likelihood phylogeny created using RAxML (Stamatakis, 2006) using a generalized time reversible model and using a rapid bootstrap calculation (-f flag set to a) and 1000 bootstrap calculations. FigTree (see foot note 4) was used to visualize the resultant best tree. To show homologies between different hsdS protein sequences, representative aligned sequences were visualized using the function msaplot in the ggtree package (Yu et al., 2017).

## Results

### Global Isolate Collection

The 132 *S. argenteus* genomes included in this study were from isolates collected from patients with *S. argenteus* infection from fourteen different countries from the years 2005–2018. The isolates were from Thailand (51%, 68), Denmark (18%, 25), Malaysia (6%, 8), China (5%, 7), Australia (3.8%, 5), United Kingdom (3%, 4), United States (3%, 4), Sweden (2.3%, 3), Singapore (1.5%, 2), and also from France, Germany, Gabon, Israel, and Japan. The source of isolates were 129 from humans, along with three from animals: one pig from China, one cow from Malaysia and one gorilla from Gabon. Clinical metadata was not available for all samples, but where present is indicated in Supplementary Table 1. Three of the four United Kingdom isolates included in this study were from Scotland and were clinically misidentified as *S. aureus* until the whole genome SNP analysis identified them as outliers and were subsequently identified as *S. argenteus* by MLST (Thomas et al., 2006) and were also crtM-negative (Chen et al., 2018). One of the Scottish isolates was from blood cultures of a patient who was severely ill and with a travel history from South-East Asia. Details of the isolates, their genomes, and their accession numbers are in Supplementary Table 1. ST assignments of genomes analyzed here were all in agreement with the original designations where published. It is important to note that the range of samples of *S. argenteus* analyzed here is liable to selection bias, since sampling strategies will vary between locations and only whole genomes that have been deposited in public databases can be analyzed.

### Core and Accessory Gene Variation

**FIGURE 1:**
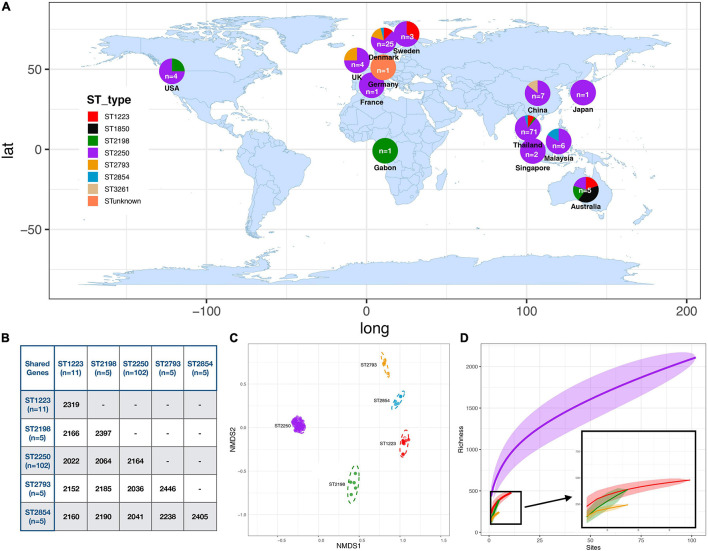
Distribution and Relatedness of Global *S. argenteus* strains **(A)** Distribution of *S. argenteus* ST within the global collection studied. The proportion of each ST within each location is shown within the overlaid pie chart. **(B)** Table showing the number of shared core genes (found in ≥99% of a specific strain) between different pairs of *S. argenteus* STs. **(C)** Non-metric Multi-dimensional Scaling (NMDS) plot for gene diversity of accessory genes within and between dominant STs, based on Jaccard distance. Each dot represents an isolate colored with its ST according to the legend in **(A)**. **(D)** Gene accumulation curves for different STs of *S. argenteus.* STs are colored according to the key in panel **(A)**. For ST2250, the Heaps co-efficient is 0.69, indicating an open genome.

We compared the genomes of these different *S. argenteus* strains to understand the variation in core and accessory gene content. *De novo* assembly of the genomes of these strains gave the genome size from 2.6 to 2.9 Mb and the GC content varied from 31.6 to 32.7% (Supplementary Table 1). The MLST scheme identified 7 distinct STs within the isolates, and one sample with indeterminate ST for which sequence quality in one of the alleles used for typing was not sufficient to classify unequivocally within a known ST group. The global collection was dominated by ST2250 (77%, 102) followed by ST1223 (8.2%, 11) along with a diverse collection of STs (ST2854, ST2198, ST2793, ST1850, and ST3261) each with fewer than 10 isolates (Figure 1A and Supplementary Table 2). The ST ST2250 is an established clade in Thailand (59%, 60/102) and also distributed globally in many eastern and European countries (Moradigaravand et al., 2017). The pig and the cow isolates belong to ST2250 whereas the gorilla isolate was ST2198. The pangenome carried a total of 5,169 genes of which 40% (*n* = 2,069) were present in 99% of our 132 genomes. The accessory genome (contained in > 5% but <99% of isolates) consists of 3,091 genes. These genes were mostly hypothetical genes, only 32% genes having functional annotations, mostly for plasmid genes or pathogenicity island genes but no specific functional pathways were identified. Core genomes of isolates (contained in >99% of isolates with 95% minimum identity) within individual clades ST2250, ST1223 and ST2198 contained 2164, 2319, and 2397 genes, respectively. Comparisons of the genes shared between 99% of the different STs are shown in Figure 1B. This shows that hundreds of the core genes of each ST were unique (defined as less than 95% identity), indicating clade-specific gene accumulations (Figure 1B). The relationship between each isolate was investigated using the non-metric multidimensional scaling (NMDS) method where pairwise genomic distances were calculated using Jaccard distances between the strains. Formation of distinct clusters for each ST confirmed that accessory genes were conserved within a ST (Figure 1C). Gene accumulation curves for the different STs of *S. argenteus* showed a steady accumulation of new genes with successive addition of genomes – for ST 2250 the Heaps law co-efficient was 0.69 (<1) indicating an open genome (Tettelin et al., 2008). This is similar to the open genome of *S. aureus* (John et al., 2019) but in contrast to the closed genome of *S. lugdunensis* (Argemi et al., 2018). A heat map showing gene presence/absence for the pangenome of our samples shows the distribution of the core and accessory genomes across the different STs (Supplementary Figure 1).

### SNP Variation and Evolution of Different Strains

**FIGURE 2:**
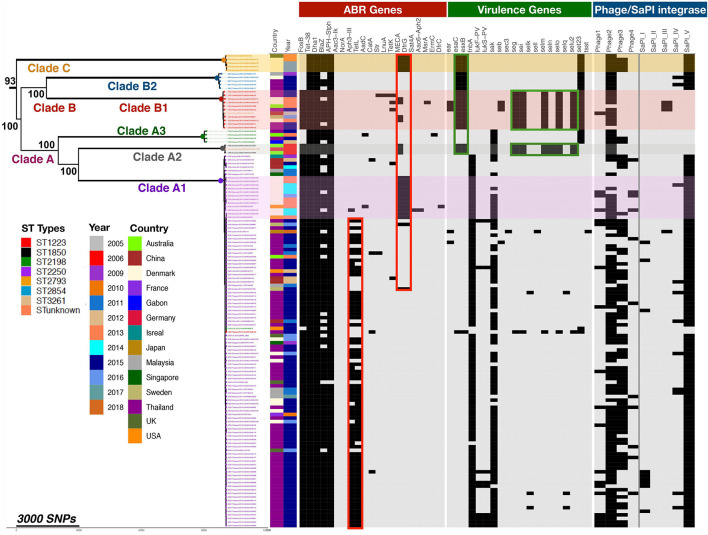
Phylogenetic relationship of global *S. argenteus* strains related to content of antibiotic resistance genes, virulence determinants, and phages. The phylogenetic tree shows relationships between the strains based on core gene SNPs. Different clades, the ST, year of isolation and country of origin are as shown. The presence of antibiotic resistance determinants, selected virulence genes, and phage integrases with SaPI island integrase present in STs are indicated to the right of the tree; presence of these factors is indicated by a black rectangle. Boxed areas highlight specific ABR (red) or virulence genes (green). Bootstrap support for the major clades is shown from 1,000 bootstrap trees. The scale bar shows the number of SNPs corresponding to branch length.

To study in more depth the population structure of *S. argenteus* and its relationship to the distribution of virulence determinants and genes conferring antibiotic resistance, we constructed a phylogenetic tree of this collection of *S. argenteus* strains using core genome SNPs and Maximum-likelihood (ML) phylogenetic analysis. Following exclusion of mobile genetic elements (MGE)s and recombination regions, the core genome was found to have a total of 34,837 SNPs in 132 genomes. This revealed three distinct clades which we have named A, B, and C, with the subdivisions as shown (Figure 2). We then overlaid the STs of the different strains on the midpoint rooted phylogenetic tree, which mostly segregated to distinct clades and subclades, but with some exceptions. Clade A1 contained the dominant ST ST2250, but also one sample of ST2198 and one of ST1223. It is not clear why these two samples segregated into this clade. Clade A2 contained both ST1850 and ST unknown, while clade B1 contained ST1223 and ST3261. We attempted to construct a time-scaled tree of the genomes to establish the evolution of the different clades over time but this was not possible as the raw sequence reads were not available for many of the sequenced strains.

### Antibiotic Resistance Genes

We determined the distribution and spread of antibiotic resistance genes within this population to determine if such genes were generally present or clade specific. Compared to *S. aureus*, antimicrobial drug resistance of *S. argenteus* is known to be lower (Chantratita et al., 2016; Aung et al., 2017). In this collection, 21 antimicrobial genes were identified and the distribution of these genes within different clades is shown in Figure 2. The *mec*A gene conferring resistance to methicillin was been identified in 14% (*n* = 14/102) of ST2250, 45% of (*n* = 5/11) ST1223 and in all five genomes of ST2793. None of the Thailand isolates have *mec*A, but it has been acquired by isolates from other geographical areas. It was most prevalent in isolates from Denmark (68%, *n* = 17/25), but also found in those from Australia (40%, *n* = 2/5), United States (40%, n = 2/4), United Kingdom (25%, *n* = 1/4) and isolates from Sweden and Germany (Figure 2). These isolates all contained a SCCmec IV element. In addition to methicillin resistance, these isolates also contained elements conferring resistant to penicillin (*blaZ*), trimethoprim (*dfrA*), and cadmium (*cadC)* (Moradigaravand et al., 2017). 50% of isolates carry the *tet*L gene mediating resistance to tetracycline, whereas 5% of isolates carry *tet*K; *tet*L was found only in the ST2250 clade (67/103, 65%). About 90% of *S. argenteus* isolates carry the fosfomycin resistance element *fos*B, which is reportedly rare in the *S. aureus* population (Fu et al., 2016b; Xu et al., 2020). The gene was highly conserved within the strains of *S. argenteus* analyzed here, with 99.4% pairwise nucleotide identity over a sequence length of 420 nucleotides. BLAST nucleotide searching in *S. aureus* genomes revealed *fosB* gene sequences with over 98% identity to the *fosB* gene in *S. argenteus* (21,609 records found in 2,199,446 total *S. aureus* nucleotide sequences in the NCBI database (0.98%) - in both organisms where complete assemblies were available, the gene was located within the bacterial chromosome. A range of *fos* genes have been described which share the common feature of inactivating fosfomycin by enzymatic opening of its oxirane ring (Castañeda-García et al., 2013). A number of subtypes with alphabetical suffixes have been described in both Gram-positive and Gram-negative bacteria, including a range of *fosB* genes identified in plasmids of *S. aureus* (Fu et al., 2016a) that differ from the chromosomal *fosB* gene. BLAST searching for these plasmid *fosB* sequences in the NCBI database found only 8 *S. aureus* records containing sequence matches with >95% identity. Representative examples of these different *fos* genes were aligned and these comparisons with a phylogenetic tree of the sequences are shown in Supplementary Figure 2. This highlights the close similarity of the chromosomal *fosB* genes of *S. aureus* and *S. argenteus*, which differ significantly from plasmid borne *fosB* genes described in *S. aureus*.

### Virulence Gene Content

**FIGURE 3:**
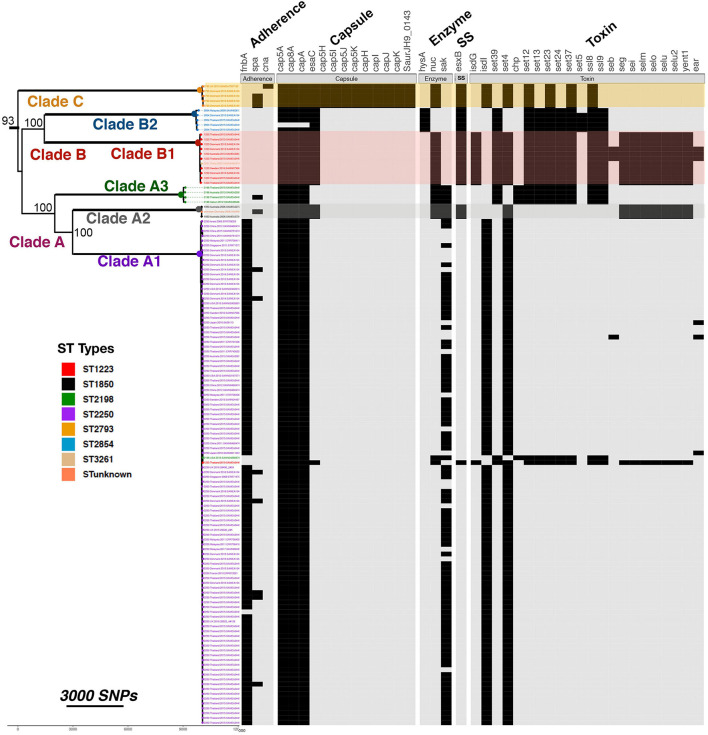
Comparison of virulence gene content in different STs. The presence and absence of specific virulence genes within each isolate are shown by black rectangles. Genes are grouped according to their broad functions as shown. SS indicates secretory system. The phylogenetic tree shows relationships between the strains based on core gene SNPs. Bootstrap support for the major clades is shown from 1,000 bootstrap trees. The scale bar shows the number of SNPs corresponding to branch length.

**FIGURE 4:**
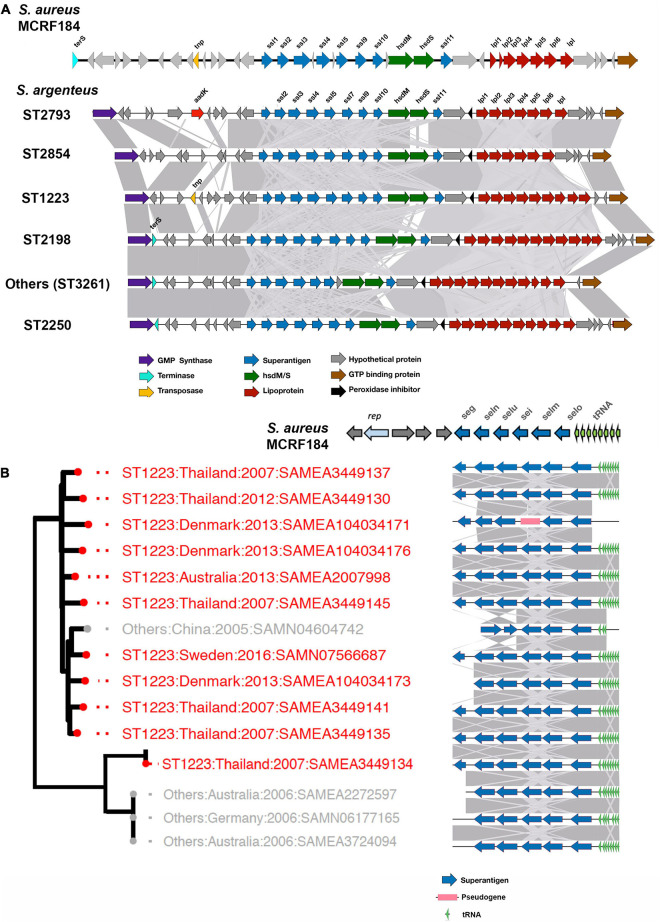
vSAα and vSAβ elements in *S. argenteus.* The figure shows the gene composition of vSAα **(A)** and vSAβ **(B)** elements in the strains of *S. argenteus* indicated. In panel **(A)**, a representative member from each ST is shown. Gene functions are color coded as indicated. The degree of similarity between the different *S. argenteus* strains is shown by the gray shading. In both panels, the arrangement of genes in the cognate elements of the MCRF184 strain of *S. aureus* is shown. *rep* is a bacterial replicase (helicase).

**FIGURE 5:**
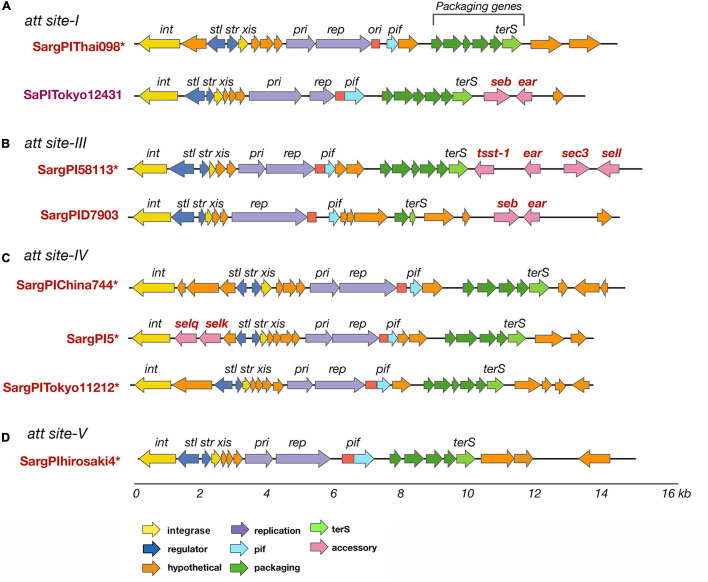
SargPI elements found at different attachment sites in *S. argenteus* isolates. SargPI elements at the different attachment sites are shown in panels **(A–D)**. * indicates a novel SargPI found within our isolates. Genes *int* and *xis* (in yellow) are excisionase; *str* and *stl* (dark blue) are transcription regulators; *pri* and *rep* (purple) are replication genes; *ori* (red) is replication origin; terS (light green) is terminase with other packaging genes (dark green); *ear, seb, sec3, sei, selq, selk, sem*, and *tsst-1* (pink) are super-antigens; *pif* (light blue) phage interference gene and hypothetical genes are in orange. The color of the genes is as shown for SaPIs [22] for easy comparison. The lower scale shows size of the SargPIs in kb.

Comparative analysis of the 132 *S. argenteus* sequences for a range of virulence factors identified a total of 213 virulence genes that differed between the different strains, as well as differing phage content (Figure 2 and Supplementary Table 3). These include bacterial toxins, cell surface proteins that mediate bacterial attachment, secretion system components, cell surface carbohydrates and proteins that protect a bacterium, and hydrolytic enzymes that may contribute to the pathogenicity of the bacterium. The average number of virulence genes in ST1223 and ST2793 was found to be significantly higher (*p* < 6.7 × 10^–9^ and *p* < 4.9 × 10^–5^, respectively) compared to ST2250 (Supplementary Figure 3). ST1223 has previously been noted to have a larger number of virulence genes (Aung et al., 2021). Substantial differences in virulence factors were found between STs (Figure 3). Adherence and secretion system genes were present selectively in all STs. However, toxin genes were more common in ST1223 compared to ST2250. These genes were mostly found in bacteriophages, genomic and pathogenicity islands (Figures 4, 5). In most cases, the virulence determinants were similar to those found in *S. aureus*.

In *S. aureus*, important virulence determinants are frequently found in genomic islands. One of these is the genomic island νSaα (Type I and Type II) associated with a cluster of staphylococcal superantigen-like (*ssl*) genes which varies considerably between different strains of *S. aureus* (Lina et al., 2004). vSaα also contains a cluster of genes encoding lipoproteins. This island was found in all the *S. argenteus* strains studied here (Figure 4A). As is the case with *S. aureus* (Lindsay and Holden, 2004; Lindsay, 2010), there is some variation within the *ssl* genes and other genes within the island. However, the overall structure was well conserved within the different strains, and there was no significant difference between different STs.

The *S. aureus* enterotoxins *seg*, *sei*, *selm*, *seln*, *selo, and selU2* are harbored in a genomic island termed νSaβ (Type I) (Omoe et al., 2005); this was also identified in *S. argenteus*, almost exclusively in clades B1 and clade A2 (Figure 2). These enterotoxins cluster together in what is termed the enteroxin gene cluster (*egc*) (Jarraud et al., 2001; Thomas et al., 2006) which is widely distributed within strains of *S. aureus*, although there is considerable allelic variation of this locus between strains (Lindsay and Holden, 2004, 2006). The *egc* locus found in the different strains of *S. argenteus* clades B1 and A2 is shown in Figure 4B. Broadly, the *egc* locus in these strains was very similar to the locus found in some strains of *S. aureus*, such as the ST45 strain MCRF184 (Aswani et al., 2019), although lacking the gene encoding a helicase (*rep)*, which is found in that strain. Collery et al. (2009) classified the locus into different types based on their gene content; the predominant type found in the *S. argenteus* strains described here is their *egc2*. However, as with *S. aureus*, there is variation; for example in the strain SAMN4604742, the *seg* gene is lost and the orientation of the *selu* and *seln* genes is reversed (Figure 4B).

There are other differences between the strains of *S. argenteus*. Genes encoding type VII secretion system extracellular proteins (*esa*C, *esx*B) were missing from clade A1 and A3. The genes encoding the PVL Panton-Valentine leucocidin (PVL), *luk*F-PV, *luk*S-PV, carried by prophages were identified in nine ST2250 isolates. The staphylokinase encoded by *sak*, which activates plasminogen to plasmin and inactivates bacterial defensins (Bokarewa et al., 2006), was found in clade A in 79% (81/102) of ST2250 and in 100% of ST2198. Fibronectin binding protein gene *fnb*A was only found in clade A1 and absent for all others. Some toxin genes were common across all clades. These were: the enterotoxin gene (*sel*X) which plays a significant role in the inhibition of host innate immune system pathways; the staphylococcal enterotoxin Y gene *(sec*Y); and antitoxin component of type-II toxin-antitoxin (TA) system genes (*maz*E, *Yef*M).

### Pathogenicity Islands

*Staphylococcus aureus* contains a wide variety of phage mobilized pathogenicity islands (Novick et al., 2010). We searched for these *S. aureus* Pathogenicity Island (SaPI)-like elements present in *S. argenteus*, as described in the Materials and Methods. New genomic islands were found that possess common features of a SaPI and very similar mosaic structure of its open reading frames (ORFs.) They have direct repeats at the both ends, along with the basic components of a SaPI: *int, stl, str, xis, pri, rep*, and *ter*S genes. The integrases (*int*) showed very high sequence similarity to SaPI integrases (>90%) and the attachment sites were similar to those of previously reported SaPIs (Novick et al., 2010). We referred to these genomic islands as SargPIs using the nomenclature previously defined (Subedi et al., 2007; Novick et al., 2010). Within our 132 *S. argenteus* isolates, 115 (86%) had at least one SargPI and 22 (17%) isolates have two of these islands each. Within these SargPIs, four novel SargPIs were identified: SargPI58113 (*att*-site III), SargPI5 (*att*-site IV), SargPIThai098 (*att*-site I), and SargPIChina744 (*att*-site IV) shown in Figure 5 and Supplementary Figures 4–7. The first pathogenicity island, SargPI58113, was identified in reference genome *S. argenteus* 58113, harboring toxin gene *tsst-1* along with *ear*, *sec3* and *sell* genes. Its integrase was 98% similar to SaPImw2 but matches 50% of SaPIbov1 (Supplementary Figure 5). One of the Thai ST2250 strains was found to have this SargPI58113 island. The second island, SargPI5, contained *selk* and *selq* toxin genes and had the integrase of SaPI5, but identical to only 30% of the island. Three of our genomes have this SargPI5 island (Supplementary Figure 6). Another novel pathogenicity island, SargPIThai098, was found in 8 of our isolates whose integrase was 88% similar to SaPI1 (Figure 5A). These islands did not harbor any known toxin genes but incorporated bacteriophage νSa1 (Φ55) (Goerke et al., 2009). The fourth island, SargPIChina744 was identified with 10% island similarity with SaPI5 in one genome without any toxin genes (Figure 5C). Previously defined pathogenicity island SargPID703 (Hansen et al., 2017) contained virulence genes *seb* and *ear*, and was present in four genomes at the *att*-site III (Figure 5B). This island is seen only in clade ST1223. We have also found some SaPIs within our isolates. SaPITokyo12431 carrying *seb* and *ear* toxin genes was present in two isolates of ST2250 (Figure 5A). SargPITokyo11212 was identified in seven genomes of ST2250 isolates, conserving 85% of the SaPITokyo11212 but having lost the enterotoxin genes *seb* and *ear* from it (Figure 5C). The island SargPIhirosaki4 was identified in three isolates of *S. argenteus* at the *att*-site V, which was 84% similar to SaPIhirosaki4 with missing toxin genes (Figure 5D).

### Type I Restriction Modification System

*Staphylococcus aureus* contains a type I restriction modification system comprising genes encoding DNA modification (*hsdM*), a restriction unit (*hsdR*), and a sequence specificity element (*hsdS*) (Waldron and Lindsay, 2006). The *hsdM* and *hsdS* encoded subunits together form a sequence specific methylase – they can also combine with the *hsdR* encoded product to form a DNAse that cleaves DNA at unmethylated sequences as specified by the *hsdS* product. In this fashion, the organism protects itself against uptake of foreign DNA, such as harmful bacteriophages. The specificity of the hsdS protein subunit is specific to particular sequence types of *S. aureus*, thus limiting horizontal transfer of genes from other strains (Cooper et al., 2017). This sequence type specificity of the hsdS protein sequence is not seen in the closely related species *S. epidermidis*, where the type I restriction modification system shows significant variation even within a single lineage (Lee et al., 2019); however, this report found that 38% of the *S. epidermidis* strains studied lacked a type I modification system. In order to understand how a type I restriction modification system might influence horizontal gene transfer in *S. argenteus* and whether this differed from *S. aureus*, we undertook a detailed analysis of the hsdS protein sequences in the sequences studied here. 121 (92%) of the 132 sequences analyzed contained at least one copy of the *hsdS* gene; of these 101 isolates had one copy [ST2250 (91 isolates), ST2854 (5 isolates), ST2198 (5 isolates)], 17 isolates had 2 copies [ST1223 (11 isolates), ST2793 (5 isolates) and ST 3261 (1 isolate)], and 3 isolates had 3 copies [ST1850 (2 isolates), indeterminate ST (1 isolate)]. Those strains without an *hsdS* gene were all members of ST2250.

**FIGURE 6:**
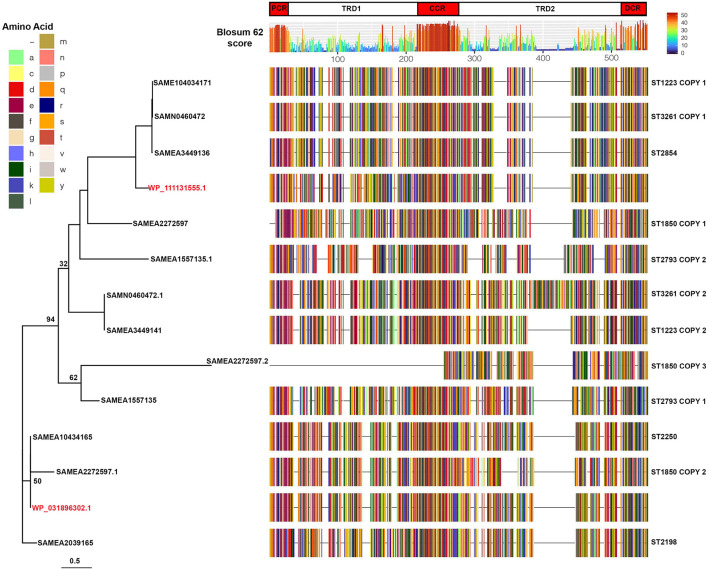
Sequence comparisons of hsdS proteins in *S. argenteus* strains. Representative sequences of the aligned hsdS proteins from the different *S. argenteus* STs are shown, colored by amino acid residue as shown in the key. Two *S. aureus* hsdS protein sequences are shown in red, labeled with their corresponding National Center for Biotechnology Information identifier. Blosum62 homology scores across all the sequences are shown above the alignment, colored as shown in the key. The domains of the hsdS protein are shown above the homology score graph – PCR, proximal conserved region; TRD1 and 2, target recognition domains 1 and 2; CCR, central conserved region; DCR, distal conserved region. The phylogenetic tree to the left shows the sequence relationships. Bootstrap support for the major branches is shown (1,000 bootstraps). The scale shows the average number of substitutions per site.

In order to determine if specific strains of *S. argenteus* had conserved type I restriction modification systems that were not shared by other strains, we carried out sequence comparisons of the hsdS proteins. The protein sequences were chosen for comparison rather than nucleotide as it is the variation in protein sequence that will dictate the specificity of the hsdS subunit and hence the target of the type I restriction modification system. Strains of the same ST had very similar hsdS proteins. Of those STs with only 1 *hsdS* gene, the hsdS protein sequences had 100% average pairwise identity for ST 2854, 99.12% for ST2198, and 99.99% for ST2250. For those strains that had more than one *hsdS* gene, the protein sequences encoded by the additional gene(s) grouped together in a given ST but were distinct from the other copy (Supplementary Figure 8). Note that some of the copies have significant deletions, e.g., copy 3 of SAMEA2272597 and SAMEA3724094. We compared the sequences of representative hsdS proteins from different STs and constructed a phylogenetic tree using RAxML. Even with 1,000 bootstraps, support for some of the branches was poor. However, the sequence comparison showed the variation in the sequences was located within the two target recognition domains of the proteins, with conservation of sequence in the proximal, central and distal regions (Figure 6). This suggests that these proteins direct distinct methylation patterns within their respective STs, and thus may serve to limit horizontal gene transfer between STs of *S. argenteus*, in much the same fashion as described for *S. aureus.*

### CRISPR/Cas Locus

**FIGURE 7:**
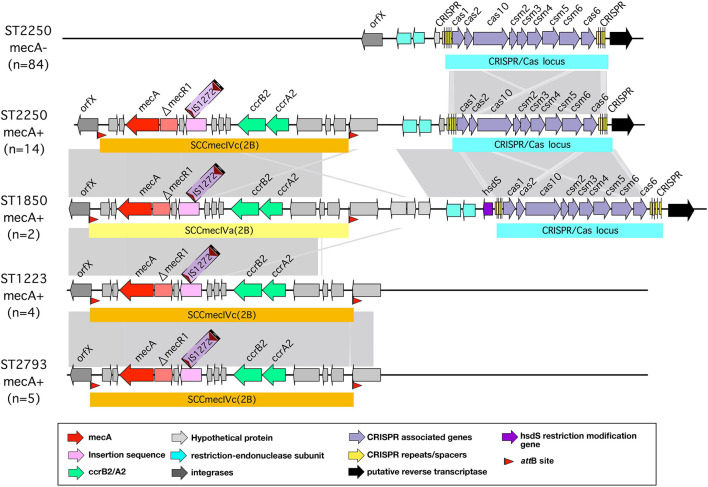
CRISPR/Cas locus in different strains of *S. argenteus*. Arrangement of the CRISPR/Cas locus within the *orfX* region of different strains of *S. argenteus* with and without the *mecA* cassette. For comparison, the *mecA* cassette in strains without the CRISPR/Cas locus are also shown.

Many bacteria also limit introduction of foreign DNA from phages or plasmids by a system characterized by clustered regularly interspaced short palindromic repeats (CRISPR) and CRISPR associated (Cas) systems. These CRISPR/Cas systems allow bacteria to capture sequences from invading phages or plasmids in heritable DNA arrays. These then are used by the Cas protein to recognize identical sequences when they are encountered on phage or plasmid invasion and to target them for degradation (Makarova et al., 2011; Rath et al., 2015). In general, few strains of *S. aureus* possess a CRISPR/Cas locus (Cao et al., 2016); previous studies have found this locus closely adjacent to the *mec* locus in MRSA + strains, some of which are prevalent in livestock (Golding et al., 2010; Cao et al., 2016). Analysis of the sequences studied here showed the presence of a type IIIA CRISPR/Cas locus found in almost all (98/102) ST2250 strains, and also in three closely related ST1850 strains. It was not present in the other STs studied. In all cases, the CRISPR/Cas element was found closely adjacent to the chromosomal locus containing the *attB* site at the 3′ end of the *rlmH* gene encoding a rRNA methyltransferase (formerly termed *orfX*) - this is the site of insertion of the *mecA* chromosomal cassette where present, as shown in strains with and without this cassette in Figure 7. Most strains with a CRISPR/Cas element had two CRISPR regions. The type IIIA CRISPR/Cas locus in these strains is syntenic with the CRISPR/Cas locus found in the livestock-associated *S. aureus* strain 08BA02176 (Golding et al., 2012).

**FIGURE 8:**
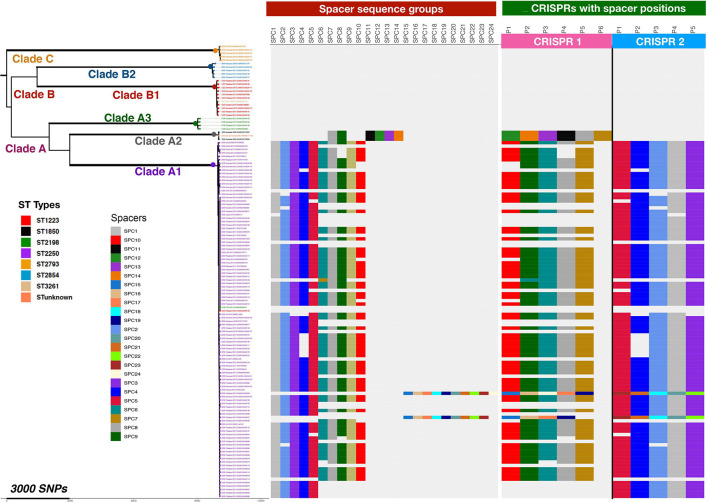
Distribution of Phage and Plasmid spacer sequences in strains of *S. argenteus.* Phage and plasmid derived spacer sequences within CRISPR/Cas repeats of different strains of *S. argenteus* are shown, grouped as defined in Supplementary Table 1 and arranged according to the phylogenetic relationships of the different strains as shown in Figure 2. The left hand panel shows the occurrence of the different sequence spacer groups in the different strains. The right hand panel shows the location of the spacers within the two CRISPR/Cas spacer groups. Spacer positions are designated P1, P2, etc, as they are found in the CRISPR/Cas locus, starting with P1 at the left hand end of the spacer regions as shown in Figure 7.

The spacer regions of the CRISPR/Cas arrays frequently contain sequences with high homology to phage and plasmid sequences that would then provide immunity to introduction of these elements into the bacterial cell. Database searching using the sequences of the spacer elements found in the *S. argenteus* CRISPR/Cas arrays revealed that they contained numerous examples of phage and plasmid derived sequences (Figure 8 and Supplementary Table 4). We grouped overlapping matches and assigned them specific group names. The distribution of the different groups within the *S. argenteus* strains is shown in Figure 8. The elements include sequences from a number of different phages known to infect *S. aureus*, as well as plasmid sequences from the pWBG plasmid series first described from remote areas of Western Australia (Shearer et al., 2011). Other sequences from Staphylococcal genomes are from spacer regions of CRISPR/Cas loci of other strains.

## Discussion

We have performed a comprehensive analysis of 132 *S. argenteus* genomes from isolates collected from fourteen countries over 13 years. Two sequence types dominate the present global collections – ST 2250 and ST 1223 – with other diverse STs in much smaller numbers. We show that there is significant variation in genetic structure within this organism, even within a specific sequence type, with differing virulence and antibiotic resistance determinants. As with the closely related *S. aureus*, we found the majority of isolates of *S. argenteus* contained at least one mobile *S. aureus* like pathogenicity island, containing a number of different virulence determinants, and identified novel elements. The strains contain strain-specific Type I restriction-modification systems, and almost all ST2250 strains contain a CRISPR/Cas system which is not found in other strains. Taken together, these findings demonstrate that *S. argenteus* has considerable genetic diversity. This may reflect different selection pressures that have allowed it to establish distinct ecological niches occupied by different STs, although neutral mechanisms such as geographical separation and genetic drift may also be important (Sheppard et al., 2018). In addition, it is important to emphasize that the collection of sequences analyzed here is subject to significant bias, since sampling strategies will vary significantly between different locations, particularly where opportunities for surveillance are limited, and resources for whole genome sequencing are limited.

The four most prevalent clades found within these isolates were ST2250, ST1223, ST2198, and ST2854. ST 2250 dominates the current global sequence collection, particularly Thailand, where it has been studied most extensively (Thaipadungpanit et al., 2015; Chantratita et al., 2016; Moradigaravand et al., 2017). However, this may not necessarily reflect its true prevalence in that region because of non-random sampling strategies. Given that *S. argenteus* is not readily differentiated from *S. aureus* by routine microbiological tests, its true global distribution remains unclear. Our study has shown distinct gene differences between the different clades, which may reflect different evolutionary pressures. Differing virulence and antibiotic resistance determinants, as well as distinct mobile genetic elements, demonstrate that this species, although only recently described, has considerable genetic diversity.

Although in general antibiotic resistance in *S. argenteus* appears less widespread than in *S, aureus*, we show here some important resistance determinants. We found that about 90% of isolates contain the *fosB* gene, which can mediate resistance to the antibiotic fosfomycin (Fu et al., 2016a). However, phenotypic resistance to this antibiotic in *S. argenteus* strains from Thailand was reported as very low (Chantratita et al., 2016). Further studies are required to establish the relationship between possession of the *fosB* gene in *S. argenteus* and phenotypic resistance to fosfomycin. Susceptibility of *S. aureus* to fosfomycin is reported to be greater than 90% in 7 of 9 studies reviewed in Vardakas et al. (2016). Fosfomycin is an old antibiotic, having been discovered in 1969, with antimicrobial activity against a wide spectrum of both Gram-positive and Gram-negative organisms. Its use in humans is limited, although it has significant potential for use as a second-line agent in treating infections due to resistant organisms, particularly in urine (Michalopoulos et al., 2011). It is also used in veterinary medicine, particularly in treating infections in chickens and pigs (Perez et al., 2014). The *fosB* nucleotide sequences were highly similar with 99.4% average pairwise identity across all the *S. argenteus* isolates, suggesting recent acquisition. Complete genome sequences were only available for a few of the isolates to allow unequivocal localization, but where a complete assembly was available, the gene was present in the genome not a plasmid and was distinct from *fosB* genes found in plasmids of *S. aureus*. The widespread presence of the resistance determinant *tetL* in isolates of *S. argenteus* was previously noted in ST2250 isolates from Thailand, apparently carried on a plasmid together with heavy-metal resistance genes and thought to be imported from livestock associated strains of *S. aureus* (Moradigaravand et al., 2017). We did not find the *tetL* gene in other sequence types of *S. argenteus*, and although predominantly present in isolates from Thailand, it was also found in some ST2250 strains from other geographical areas, including Denmark, United States, Malaysia and Singapore. Given the bias toward strains from Thailand in the collection of sequences analyzed here, it is difficult to determine the origin of the *tetL* gene within *S. argenteus.* Geographical limitation of antibiotic resistance is, however, seen with the distribution of the *mecA* element, mediating methicillin resistance. This determinant was not found in ST2250 isolates from Thailand, but was present in ST2250 isolates from Denmark and the United States, as well as in ST2793. The element belonged to the SCC*mec*IV complex in all the strains where present. MRSA strains are common in pigs in Denmark; the *mec* element in these strains is typically SCC*mec*Vc (Sieber et al., 2018). Whether the *mecA* element found in Danish strains of *S. argenteus* originated in strains of *S. aureus* within pigs will require further study of isolates from both humans and pigs. Taken together, however, the data contained in this report is certainly consistent with gene flow of antibiotic resistance elements from livestock into human strains.

The presence of different pathogenicity islands within specific STs suggests specific survival advantage of these islands within specific STs. Moreover, gene variation analysis shows the accumulation of different virulence factors in different clades over time. The analysis shows that evolution of ST1223 is separate from that of ST2250. For example, ST1223 carries the gene for a siderophore surface protein (*isd*B) which has been lost during adaptation of clade ST2250. However, ST2250 has acquired prophages harboring PVL genes associated with skin and soft-tissue infections and increased morbidity. Movement of genomic islands in and out of the clades results in success of one clade over another. Novel pathogenicity islands identified in ST2250 and associated multiple phages may be the reason for the clade being successful in its spread compared to ST1223. A number of specific virulence genes encoding enterotoxins are found predominantly in ST1223, such as *sei, seg, selm, selu2*, and *set23.* These genes are typically clustered together in *S. aureus* as a cluster known as the *egc* locus, which has undergone significant rearrangement in different strains (Jarraud et al., 2001; Thomas et al., 2006). In the ST1223 strains analyzed here, we also found clustering of these genes as shown in Figure 4A. In their original description of a *S. argenteus* as a divergent CC75 clade, Holt et al. (2011) noted the presence of the νSaβ island with an enterotoxin gene cluster similar to that found in non-CC75 *S. aureus* lineages, arguing that this locus is ancestral to the separation of *S. argenteus* from *S. aureus.* The similarly of this locus in ST1223 and related strains we describe here support this view, although it has been largely lost in other lineages, particularly in ST2250 strains. This suggests a specific selection pressure for retention in the ST1223 and related strains that reflects differing ecological niches – the apparent association of ST1223 strains with food poisoning (Wakabayashi et al., 2018) suggests that gastrointestinal spread of this strain may dominate compared to other mechanisms found in non-ST1223 strains. ST1223 has previously been noted to have a larger number of virulence genes (Aung et al., 2021). Further characterization of the disease associations of the different *S. argenteus* strains will clarify these differences.

A type I restriction modification system is widespread in the *S. argenteus* strains studied here. As is the case for *S. aureus*, a given ST of *S. argenteus* has essentially identical hsdS proteins which differ from other STs. This may limit the ability of specific STs to acquire genes by horizontal gene transfer from other STs or indeed strains of *S. aureus*. Despite this potential barrier to horizontal gene flow, *S. aureus* does demonstrate evidence of such transfer (Lindsay, 2010) so it is clearly not an absolute barrier, and the presence of different phages and SAPIs within *S. argenteus* suggest that is also the case in this species. We also found the presence of a CRISPR/Cas locus with captured phage and plasmid sequences found in virtually all of the ST2250 strains. These data strongly suggest that this CRISPR/Cas locus has been active in capturing DNA from relevant phage and plasmid sources and thus providing those strains with immunity from subsequent phage attack or from introduction of plasmids from other staphylococcal strains. The presence of this locus selectively in ST2250 strains suggests it confers a fitness advantage to this ST. Resisting phage lysis is clearly a selective advantage, but it is not clear why the ST2250 strains in particular have acquired the CRISPR/Cas locus and why other STs of *S. argenteus* and most *S. aureus* lack this system. Strains lacking the CRISPR/Cas locus do not have significantly more integrated phages, suggesting this system is not pivotal in resisting phage integration – the type I restriction modification system will also act to limit integration of phages from different STs. Recent work has highlighted additional functions of the CRISPR/Cas locus in virulence (Louwen et al., 2014; Solbiati et al., 2020); whether such a role has been important in the acquisition of CRISPR/Cas loci in *S. argenteus* strains will require further experimental work.

In summary, the findings reported here show that although *S. argenteus* shares many features with *S. aureus* it also has some distinct features, and significant genomic variability between different STs. Increasingly, *S. argenteus* is recognized as an invasive human pathogen, much as *S. aureus*. Expert microbiological society opinion in Europe has proposed not distinguishing between *S. aureus* and *S. argenteus* for routine reporting purposes, although if laboratories are able to make the distinction, reporting should explicitly state that *S. argenteus* is a member of the *S. aureus* complex (Becker et al., 2019). A recent paper from North America stressed the importance of clinical laboratories differentiating the two species from a research and surveillance perspective, but with the same precautions on specific wording in laboratory reports to capture the close link between *S. argenteus* and *S. aureus* (Eshaghi et al., 2021). The data presented here shows that *S. argenteus* does have some genomic features that differ from *S. aureus*, and better correlations with specific clinical syndromes and outcomes would certainly require more robust differentiation between the two species in clinical practice. Future acquisition of whole genome sequences of *S. argenteus* will add to the analysis conducted here and improve our understanding of this organism.

## Data Availability Statement

The authors confirm all supporting data, code and protocols have been provided within the article or through Supplementary Data Files. Accession numbers for all sequences reported here are contained in Supplementary Table 1.

## Ethics Statement

Ethical review and approval was not required for the study on human participants in accordance with the local legislation and institutional requirements. Written informed consent for participation was not required for this study in accordance with the national legislation and the institutional requirements.

## Author Contributions

TE, CG, SF, AL, and MH: conceptualization. TE, CG, SF, and MH: methodology. CG, SF, and TE: analysis. All authors: writing, contributed to the article and approved the submitted version.

## Conflict of Interest

The authors declare that the research was conducted in the absence of any commercial or financial relationships that could be construed as a potential conflict of interest.

## Publisher’s Note

All claims expressed in this article are solely those of the authors and do not necessarily represent those of their affiliated organizations, or those of the publisher, the editors and the reviewers. Any product that may be evaluated in this article, or claim that may be made by its manufacturer, is not guaranteed or endorsed by the publisher.

## Footnotes

1
https://github.com/tseemann/mlst

2
https://github.com/tseemann/shovill

3
https://cran.r-project.org/

4
http://tree.bio.ed.ac.uk/software/figtree/

5
https://github.com/weizhongli/cdhit

6
http://www.ncbi.nlm.nih.gov/
