# Supplementary material for: Genomic Analysis of Global Staphylococcus argenteus Strains Reveals Distinct Lineages With Differing Virulence and Antibiotic Resistance Gene Content
Source: Front Microbiol. 2021 Dec 2;12:795173. doi: 10.3389/fmicb.2021.795173 (PMC8677677; doi:10.3389/fmicb.2021.795173)
Supplement: Supplementary file 1 [file Data_Sheet_1.zip › Supplementary Tables S2-S3 and Figures S1-S8.pdf]

Supplementary Material:

Table S2: Absolute count of strain distribution over different countries. Total numbers of each ST are shown in parentheses.

	ST1223 (11)	ST1850 (2)	ST2198 (5)	ST2250 (103)	ST2793 (5)	ST2854 (5)	ST3261 (1)	STunknown (1)
Australia	1	2	1	1	0	0	0	0
China	0	0	0	6	0	0	1	0
Denmark	3	0	0	17	4	1	0	0
France	0	0	0	1	0	0	0	0
Gabon	0	0	1	0	0	0	0	0
Germany	0	0	0	0	0	0	0	1
Israel	0	0	0	1	0	0	0	0
Japan	0	0	0	1	0	0	0	0
Malaysia	0	0	0	5	0	1	0	0
Singapore	0	0	0	2	0	0	0	0
Sweden	1	0	0	2	0	0	0	0
Thailand	6	0	2	60	0	3	0	0
UK	0	0	0	3	1	0	0	0
USA	0	0	1	3	0	0	0	0

Table S3: Phages and SargPI count in STs. Total numbers of each ST are shown in parentheses.

	ST1223 (11)	ST1850 (2)	ST2198 (5)	ST2250 (103)	ST2793 (5)	ST2854 (5)	ST3261 (1)
Phage1	0	0	1	18	0	0	0
Phage2	10	2	7	85	5	3	1
Phage3	3	0	1	61	2	3	0
Phage4	1	1	0	9	0	0	0
SaPI-I	0	0	0	9	0	0	0
SaPI-II	0	0	0	0	0	0	0
SaPI-III	1	0	0	4	0	0	0
SaPI-IV	1	0	0	9	0	0	1
**SaPI-V**	7	2	1	91	4	5	1

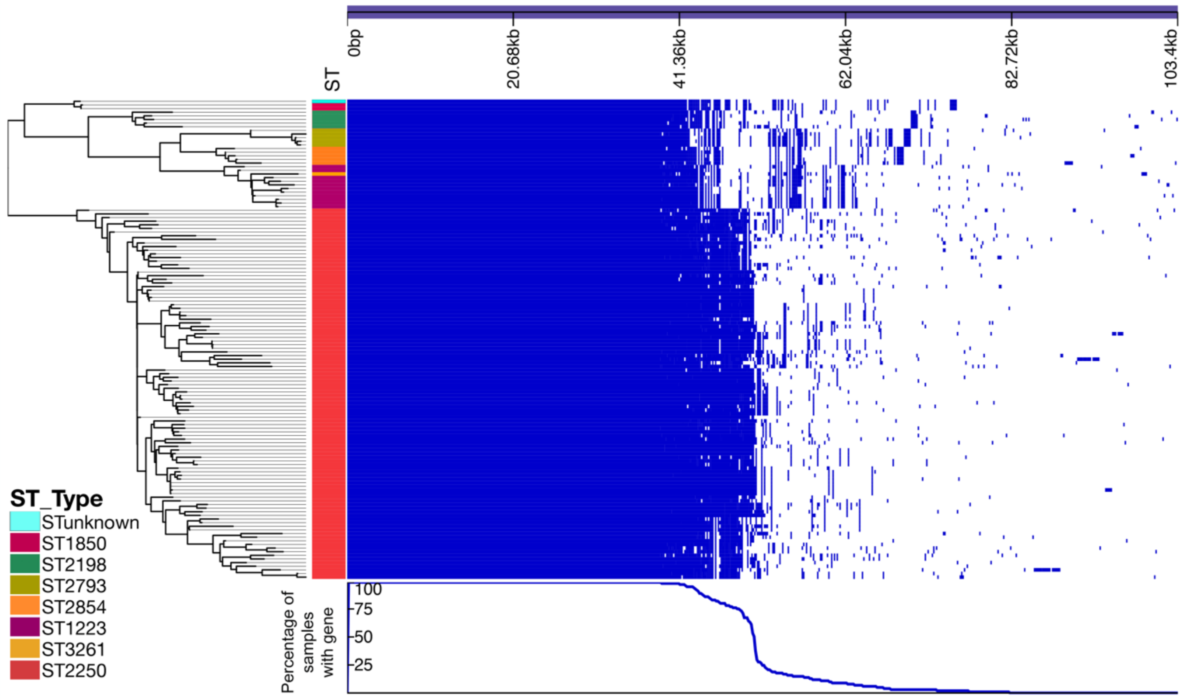

Supplementary Figure S1

Heat map showing presence/absence of genes in the pangenome ordered by the percentage of samples containing a given gene, from left to right from high to low. The tree to the left of the heatmap is based on binary gene presence/absence. The graph below the heatmap shows the percentage of the samples containing the given gene. STs of the different samples are colour coded as indicated.

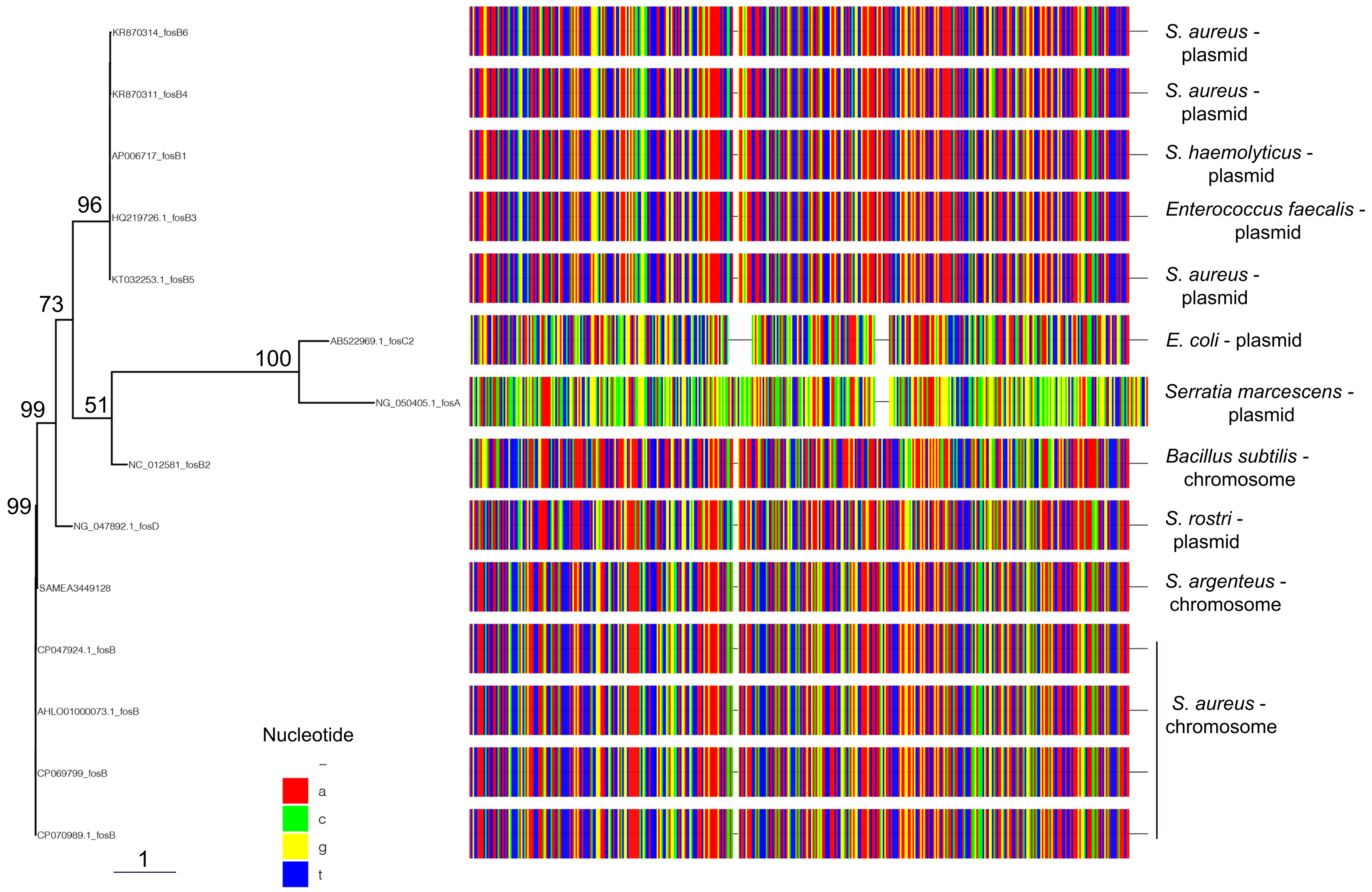

Supplementary Figure S2

Sequence alignment of *fos* genes from *S. argenteus* isolate SAMEA3449128 with other *fos* genes from other bacteria as indicated, identified by their National Center for Bioinformatics Information reference number. Phylogenetic relationships between the sequences are shown to the left as determined by RAxML with 1,000 bootstraps. Bootstrap support for branches is shown on the tree. Nucleotides are coloured as indicated. The tree scale bar indicates the length corresponding to an average of 1 nucleotide substitution per site.

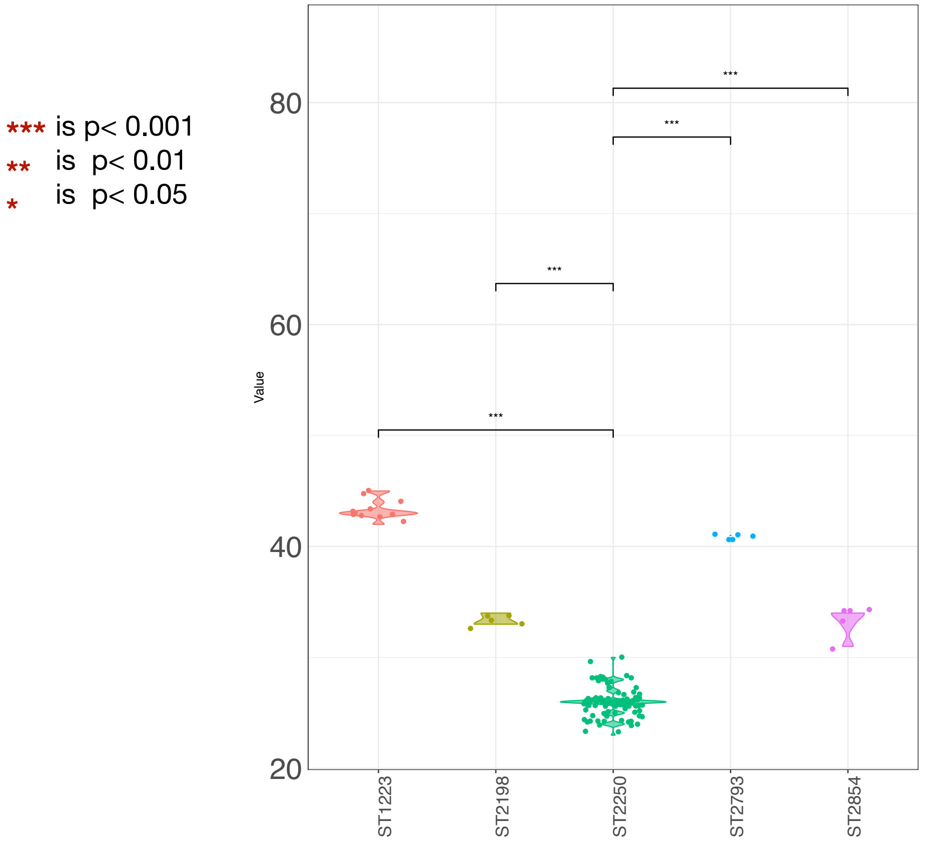

Figure S3: Virulence gene count between dominant lineages with 90% blastn identity cutoff. The interquartile range is shown shaded. The Mann Whitney test significance values compared to ST2250 are shown with Bonferroni corrected p-values. ST1223 and ST2198 showed significantly higher virulence compared to global strain ST2250.

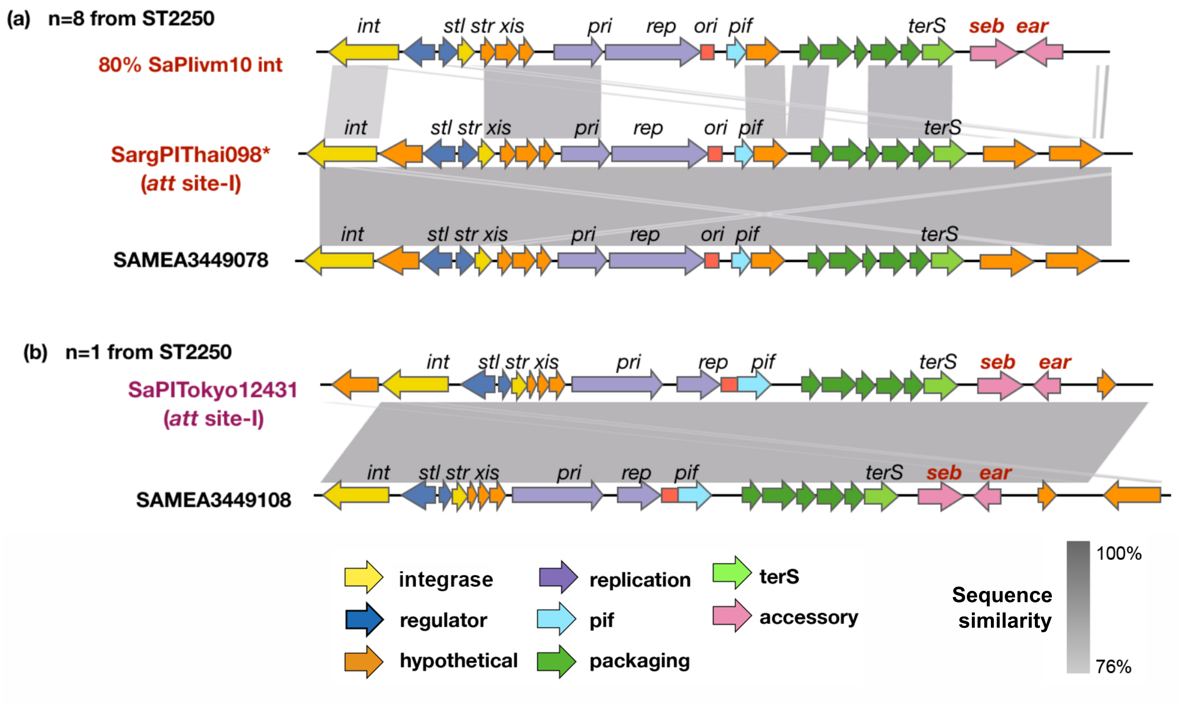

Figure S4 : SargPI elements found at *att*-site-I in *S. argenteus* isolates. Abbreviations are as in the legend to Figure 5.

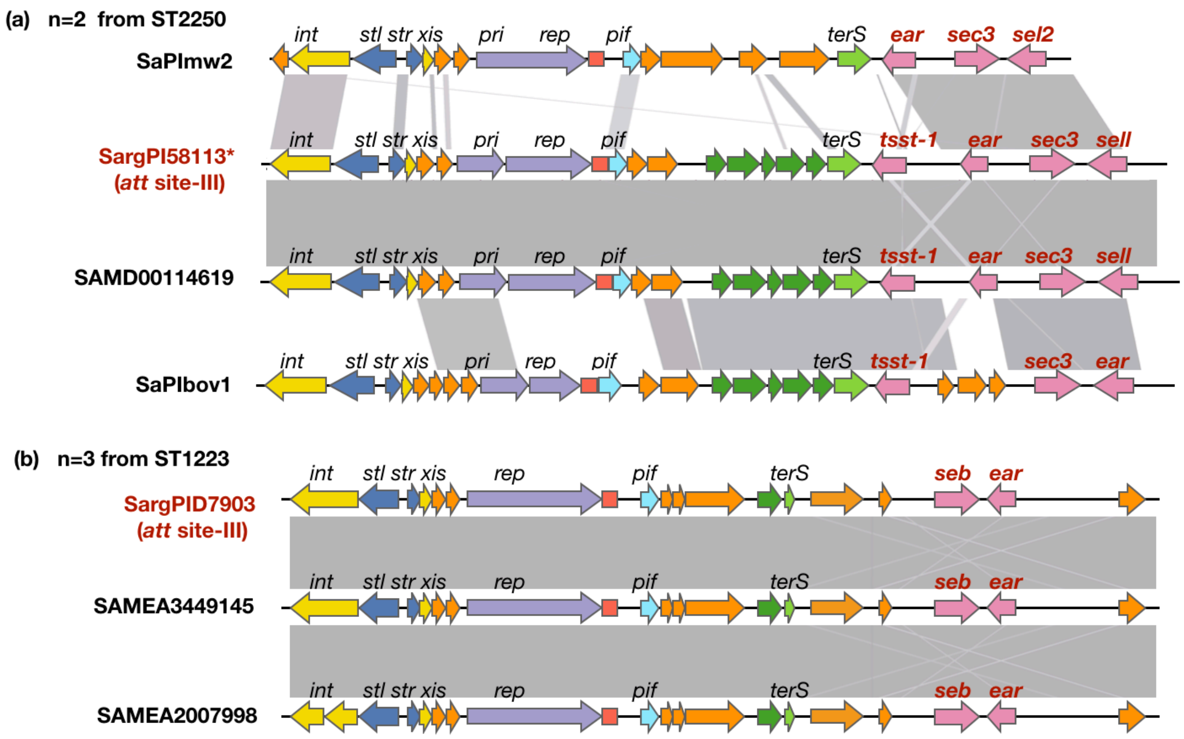

Figure S5 : SargPI elements found at *att*-site-III in *S. argenteus* isolates. Abbreviations and colour coding are as in the legend to Figure S4.

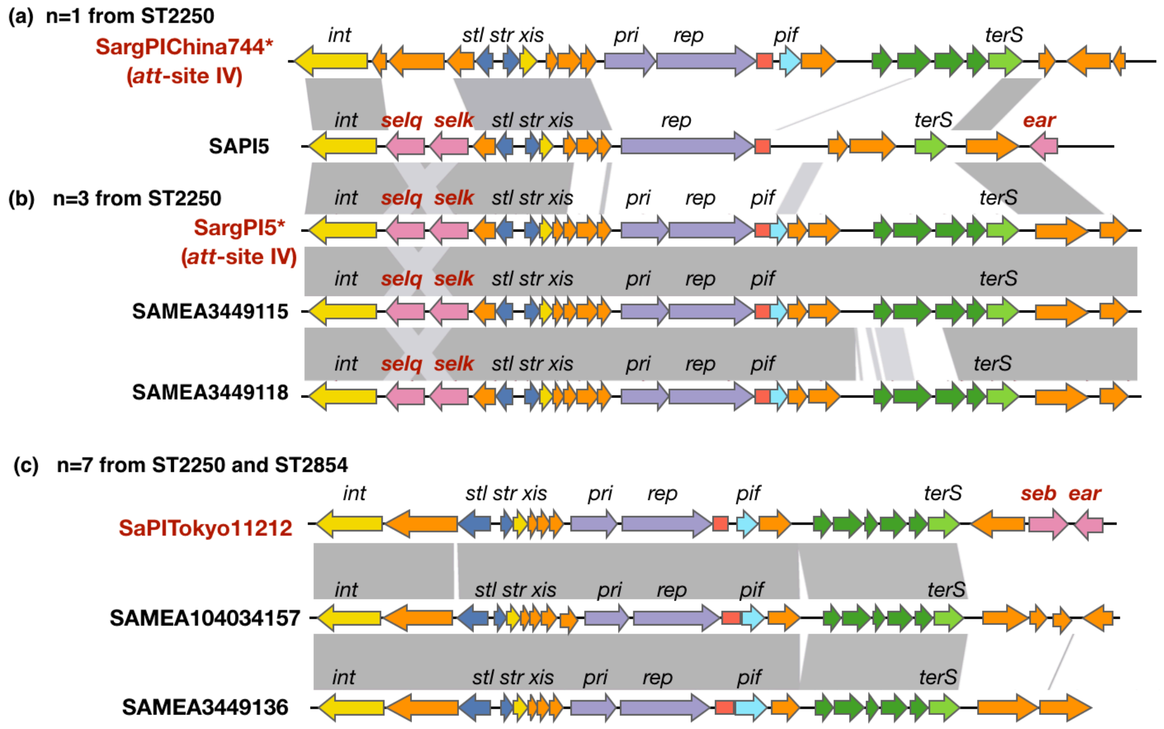

Figure S6 : SargPI elements found at *att*-site-IV in *S. argenteus* isolates. Abbreviations and colour coding are as in the legend to Figure S4.

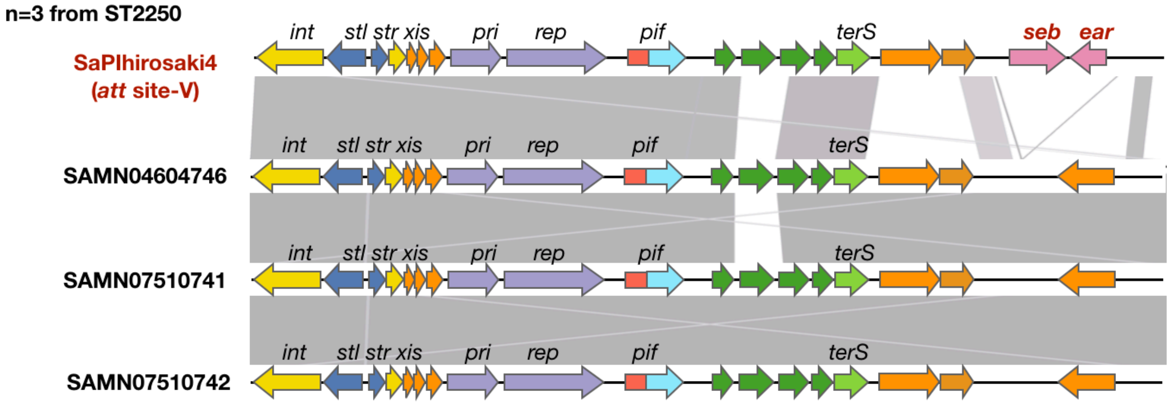

Figure S7 : SargPI elements found at *att*-site-V in *S. argenteus* isolates. Abbreviations and colour coding are as in the legend to Figure S4.

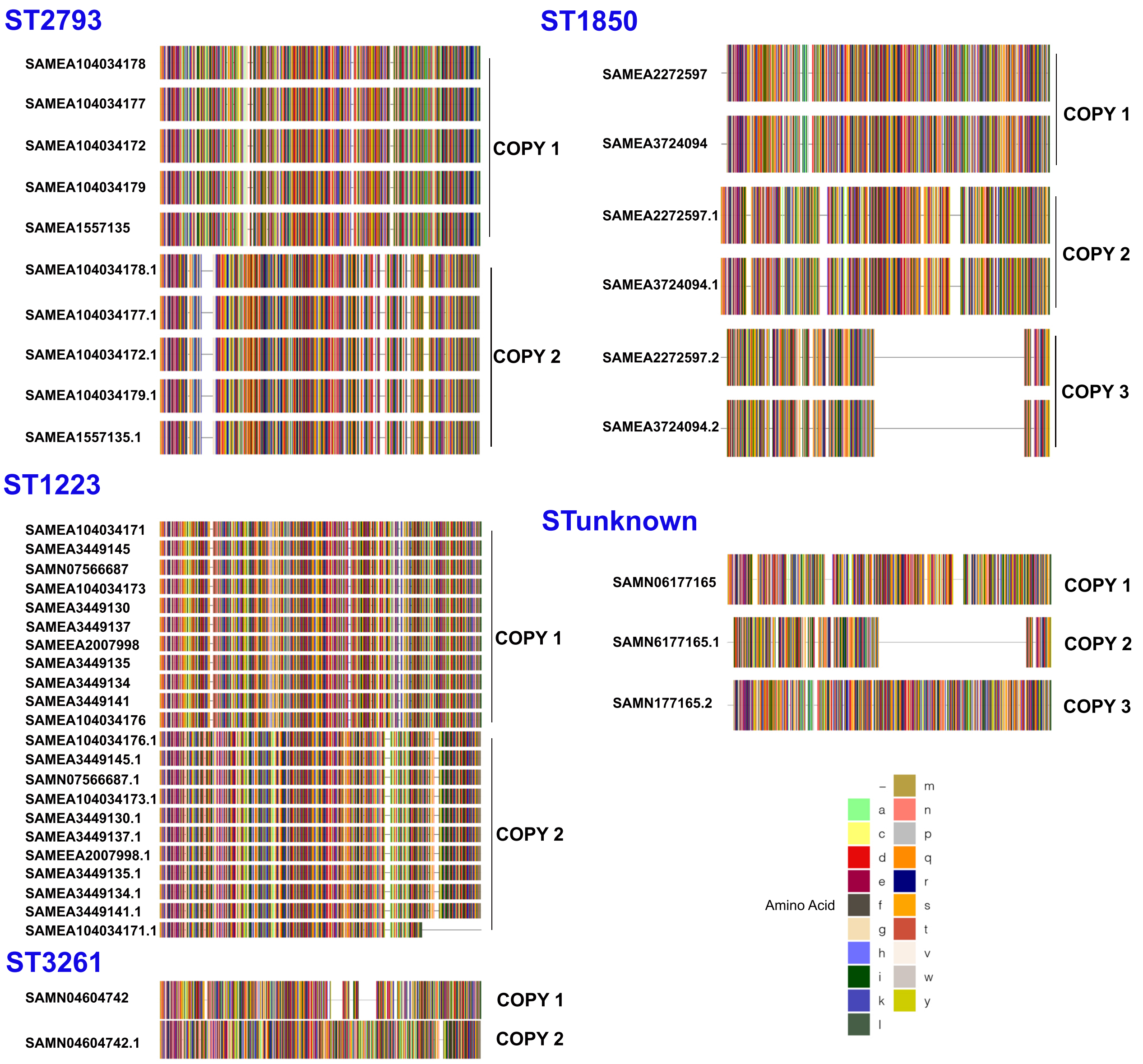

Figure S8. Multiple sequence alignments of different hsdS proteins from *S. argenteus* isolates grouped according to the different STs. Multiple copies are as indicated. Amino acids are coloured according to the key.
